# Affective Health Messages for Adolescents: Protocol for a Scoping Review

**DOI:** 10.2196/78927

**Published:** 2026-03-02

**Authors:** Siv Hilde Berg, Raul Ferrer-Conill, Kristine Stifjell, Kristin Hjorthaug Urstad, Jo Røislien, Lisbet Fjæran

**Affiliations:** 1 Department of Quality and Health Technology, Faculty of Health Sciences, University of Stavanger, Innovasjonsparken i Stavanger, Bygg i3, Stavanger, 4021, Norway, +47 90022463; 2 Department of Media and Social Sciences, Faculty of Social Sciences, University of Stavanger, Stavanger, Norway; 3 Department of Community Medicine, UiT The Arctic University of Norway, Tromsø, Norway; 4 Department of Public Health, Faculty of Health Sciences, University of Stavanger, Stavanger, Norway; 5 Department of Safety, Economics and Planning, Faculty of Science and Technology, University of Stavanger, Stavanger, Norway

**Keywords:** health communication, adolescence, affect, decision-making, health behavior, framing, narratives, emotional appeals

## Abstract

**Background:**

Affect plays a pivotal role in capturing people’s attention with health messages that influence audiences’ perceptions, attitudes, and, ultimately, health-related behavior. Adolescents are notably sensitive to affective cues in communication. However, there is a lack of reviews synthesizing the evidence examining the role of affect in health messaging targeted at adolescents.

**Objective:**

The aim of this scoping review is to systematically map and synthesize the empirical studies examining the role of affective health message content targeted at adolescents and the associated communication outcomes.

**Methods:**

We will conduct our review in accordance with the PRISMA-ScR (Preferred Reporting Items for Systematic Reviews and Meta-Analyses extension for Scoping Reviews) checklist. We will include empirical studies that examine health messages directed at adolescents from formal or informal sources, as well as studies using research-based experimental designs. Only studies focusing on public communication through mediated communication (eg, social media, the internet, and television) will be included. To be eligible, studies must explore message content targeted at adolescents’ emotions. We will include studies reporting on communication outcomes in the context of urgent or nonurgent health issues. Only English-language, peer-reviewed empirical studies published in 2014 or later will be included. Data from eligible studies will be extracted into a matrix, summarized in tabular form, and synthesized using a descriptive qualitative analysis.

**Results:**

As of May 2025, we have searched the Embase, Ovid MEDLINE, and PsycInfo databases and screened 29 reviews, which has resulted in 1856 unique hits. Scopus will be added to the search. Screening and full-text assessment will be conducted in 2026.

**Conclusions:**

We anticipate that this scoping review will provide insights into how adolescents behave, form judgments, and make decisions in response to health messages targeted at their emotions. Additionally, the review will conclude by outlining the research gaps and offering recommendations for future studies.

## Introduction

The affective-persuasive turn in digital communication is dominated by rapid, emotionally charged messaging across multiple digital platforms [1]. Affect plays an imperative role in designing content that captures people’s attention; elicits people’s emotions; fosters engagement; and, ultimately, persuades people to change their behavior [1,2]. Persuasion theory proposes that the content of a message shapes its emotional tone, which influences how deeply the audience processes the information [3]. Within this framework, message content refers to what the message communicates and includes, among other things, emotional appeals, framing, narrative elements, and the way in which the arguments are presented [3].

However, the literature lacks a comprehensive overview of how messages targeted at adolescents affect influence health decision-making and behavior within the field of health communication. The role of affect in judgment, decision-making, and behavior [4] is not sufficiently explored in research on public health communication [5]. The gap between theory and empirical application highlights the need for research that connects conceptual models, empirical studies, and real-world health communication practices.

Adolescents are notably receptive to emotionally charged health messages [5]. Adolescence represents a key developmental period marked by emotional, cognitive, and social changes. During this period, extensive remodeling of the brain’s dopaminergic system occurs, leading to increased risk-taking and reward-seeking behaviors, particularly in the presence of peers [6]. These behaviors are largely driven by changes in the brain’s socioemotional system, which matures earlier than the cognitive control system responsible for self-regulation [6]. This heightened emotional sensitivity is further amplified by the pervasive influence of social media and digital communication in adolescents’ daily lives. Digital interactions on social media can intensify affective polarization [7] and significantly impact adolescents’ emotional well-being—both positively and negatively [8,9]. Moreover, existing studies rarely take account of the rapidly changing digital environments in which adolescents operate, raising concerns about the relevance of findings predating the social media era.

Increased sensitivity to emotional cues in communication, combined with massive exposure to digital information and the fact that the contingencies for health decision-making are different for adolescents than for their older counterparts and the population at large, highlights the need for better insight into the role of affect in health communication targeted at adolescents. Despite its importance, there is a lack of reviews synthesizing evidence on the role of affect in health message content targeted at adolescents. Existing systematic reviews have primarily focused on messages on specific health issues targeted at adolescents and young adults, such as messaging strategies for preventing and ceasing e-cigarette use [10], mental health awareness campaigns [11], vaccine coverage and timeliness [12], and the promotion of healthy lifestyle behaviors [13]. Systematic reviews have also examined the emotional impact of social media and digital technology [9,14]. Thus, much of the research reviewed is issue specific and lacks integration across health domains.

A generic and broader literature synthesis examining systematically how affective message content—originating from both formal (eg, public health campaigns) and informal (eg, social media) sources—is linked to communication outcomes remains lacking. Such evidence includes a wide variation in methodological designs and outcomes and crosses boundaries among public health, health communication, emotion science, and health decision-making, among others. The lack of a common framework limits comparability across studies and makes it difficult to conduct meta-analyses and assess which strategies are most effective in different contexts. However, the scoping review methodology is particularly useful in this matter to synthesize evidence in a systematic manner and identify knowledge gaps and the scope of a body of literature with different methodologies [15].

This scoping review aims to fill these current knowledge gaps related to lack of literature reviews on the use and outcomes of affective message content in health communication targeted at adolescents by systematically mapping empirical studies. This review is guided by the following research questions: what empirical literature exists regarding health message content targeted at adolescent emotions, and what are the associated communication outcomes?

## Methods

This study is a scoping review, supporting a systematic approach to data identification, extraction, and synthesis [16]. We will conduct our review following guidance for systematic scoping reviews in health care [16] and the PRISMA-ScR (Preferred Reporting Items for Systematic Reviews and Meta-Analyses extension for Scoping Reviews) checklist (Checklist 1) [17].

### Eligibility Criteria

#### Population, Concept, and Context Framework

The population, concept, and context approach was used to specify our rationale and eligibility criteria [17].

##### Population

We will include studies in which most of the sample are adolescents (aged 10-24 years) and exclude studies that primarily sample other age groups [18].

##### Concept

###### Overview

**Figure 1.**
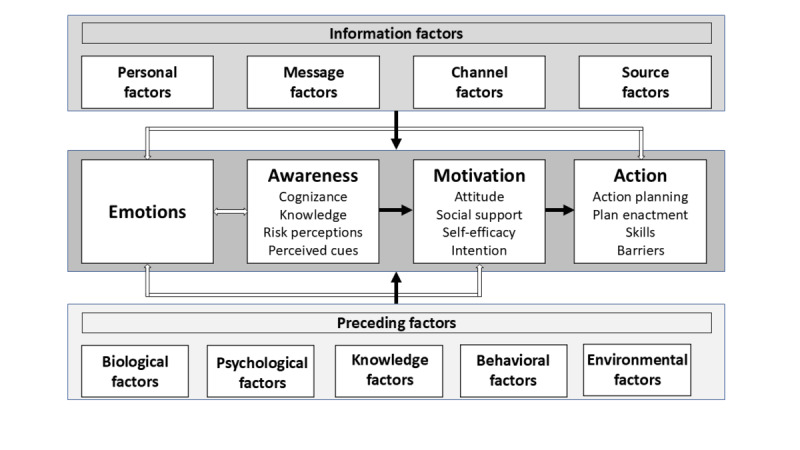
Revised integrated change model.

The conceptual criteria are organized according to the revised integrated change model [19] (Figure 1). The revised integrated change model outlines information factors (personal factors, message factors, channel factors, and source factors), communication outcomes (emotions, awareness, motivation, and action), and preceding factors. The model assumes that the communication outcomes depend on the 2 determinants: information factors and preceding factors.

###### Source Factors

We will include studies of public health messages from formal sources (eg, public health authorities or the World Health Organization or researcher-designed messages in experimental studies), informal sources (eg, influencers, advertisements, and social media), or both. We will exclude studies of personal communication (eg, communication between patients and health care professionals) and interventions that include multiple components beyond the public health messages (eg, group-based patient education interventions).

###### Channel Factors

We will include studies on diverse modes of communication, which include but are not limited to web-based information, social media, television, newspapers, video, and texts.

###### Message Factors

In this review, we will include studies that involve affective message content. Affect is defined by Lerner et al [4] as “unspecified feelings; the superordinate umbrella of constructs involving emotion, mood, and emotion-related traits.” A precise definition concerning affect in communication does not exist [20]. We understand affective message content in this context as message-level features (message factors) that have been shown to influence the recipients’ feelings and emotions as opposed to and/or next to influencing the recipients’ thoughts and beliefs [20]. Affective features of message content include emotional appeals (eg, fear, humor, and empathy), framing (eg, gain vs loss framing), narrative elements (eg, storytelling and personal experiences), and the way in which arguments are presented persuasively (eg, emphasizing severe health risks and using moral appeals) [3].

###### Communication Outcomes

We will include studies reporting on communication outcomes in a broad manner related to emotions (eg, trust and specific emotions), awareness (eg, risk, perception, knowledge, awareness, bias, and heuristics), motivation (eg, attitudes and self-efficacy), and action (eg, behavioral intentions, susceptibility to behave, and self-reported behavior and actions). We will exclude outcomes related to predisposing factors, that is, behavioral factors (eg, lifestyles), psychological factors (eg, personality and message memory), knowledge factors (eg, health literacy and knowledge), biological factors (eg, gender), social and cultural factors (eg, the price of cigarettes and policies), and information factors (the quality of messages and the channels and sources used) [19].

### Context

We will include communication outcomes related to both urgent (eg, choosing a cancer treatment) and nonurgent (eg, deciding to quit smoking) health issues or risks. We will include studies in the health context concerning adolescents, which includes communication of health risks or issues and medical decision-making regardless of the geographical location and culture of the receiver. We will exclude judgment and decision-making in nonhealth contexts or regarding nonhealth risks and issues.

### Types of Sources of Evidence

English-language peer-reviewed empirical studies will be included irrespective of data collection method. Given the rapidly evolving impact of the digital society and social media in the last decade, we will include only literature published during or after 2014. Commentaries, reviews, opinion pieces, or other papers not reporting primary empirical research will be excluded.

### Search Strategy and Information Sources

To identify potentially relevant documents, the following bibliographic databases will be searched: Embase (1974-April 2025), Ovid MEDLINE (1946-April 11, 2025), and PsycInfo (2002-April 2025). We will not undertake reference screening or contact paper authors. The search strategy was drafted by an experienced librarian and SHB, refined, and, finally, validated by the librarian. We combined search terms identified in the literature with MeSH (Medical Subject Headings) terms and proximity operators. The full electronic search strategy is presented in Multimedia Appendix 1.

We will search using the following terms: “dual process theor*,” “dual process model,” “risk as feeling,” “heuristic cues,” “heuristics,” “affect heuristic*,” “appraisal theor*,” “cognitive appraisal*,” “cognitive-experiential self-theory,” “somatic marker,” “self-efficacy,” “intuitive-experiential,” “analytical-rational thinking,” “system 1 thinking,” “system-1 thinking,” “information processing theor*,” “framing*,” “frame,” “narrative,” “empath*,” “message feature*,” “valence,” “message appeal*,” “negative appeal*,” “negative emotion*,” “positive appeal*,” “positive emotion*,” “value approach,” “presentation mode or communication mode*,” “emotions,” “affect,” “fear,” “anger,” “hope,” “shame,” “self-efficacy,” “digital communication,” “health message*,” “social media,” “health communication,” “health promotion,” “health education,” “consumer health information,” “behavior,” “health behavior,” “decision making,” “judgment,” “judgement,” “behaviour*,” “teen*,” “young people,” “young person*,” “youth or adolescen*,” “high school*,” “health*,” and “medical*.”

No filters or limits will be added to the initial literature searches regarding language and peer review. Filters will be added for publication date range (January 2014-present). No searches of gray literature will be conducted due to feasibility constraints and the need for quality assurance as gray literature often lacks peer review. The final search results will be exported into EndNote (Clarivate Analytics), and duplicates will be removed by SHB. All records will thereby be uploaded to Rayyan (software for intelligent systematic review; Qatar Computing Research Institute) for further manual removal of duplicates. In addition, manual searches will be conducted of reviews, which will be screened by 2 reviewers for primary studies fulfilling the eligibility criteria. All identified records will be logged and reported in the PRISMA-ScR flow diagram. The list of screened reviews will be provided as an appendix.

### Selection of Sources of Evidence

We will create 3 screening teams (SHB and LF, KHU and JR, and KS and RF-C) to screen titles and abstracts from the databases using the eligibility criteria outlined above and produce a list of articles to be assessed in full text. We will use Rayyan to support the screening and inclusion process of eligible studies. Within each team, members will be blinded to each other’s decisions during the screening process to minimize bias [16]. Dual independent screening will be used at both the title and abstract and full-text stages, with discrepancies resolved through discussion or a third party. The eligibility criteria will be pilot-tested by independently screening 20 articles, and the criteria will be subsequently refined based on feedback from the authors. The authors will divide the final list of articles between them and screen the articles in full text. The final list of included papers will be validated by SHB. Any uncertainties regarding inclusion or exclusion will be discussed within each team, and disagreements will be resolved through discussion with the first author (SHB).

### Data Charting Process

Following the guidance for conducting scoping reviews [16], all authors will extract the data from the included articles into a matrix ahead of synthesis. The extracted data will include (1) author names, (2) year of publication, (3) country of origin, (4) aims and research questions, (5) study design, (6) sample characteristics, (7) type of affective feature in the communication content, (8) health communication topic, and (9) key outcomes related to the research objectives. The matrix will be tested by extracting data from 1 paper per reviewer, which will then be validated in collaboration. The extracted data will be condensed and displayed in tables, and the content will be validated by SHB.

### Synthesis Method

A narrative synthesis will be applied to the results—an approach to synthesis of findings using words and text to summarize and explain the results [21]. In the context of a scoping review, narrative synthesis addresses a broad range of questions not limited to the effectiveness of specific interventions [21].

Data synthesis will follow the 4-stage narrative synthesis approach proposed by Whittemore and Knafl [22]. The first stage of analysis consists of data reduction, whereby the results relevant to the review question will be condensed and categorized according to 3 predetermined themes and categories (Table 1).

**Table 1.** Predetermined themes and categories.

Theme	Predetermined categories
Study characteristics	Year of publication Country of origin Aims and research questions Study design Sample characteristics Health communication topics
Type of affective feature in the communication content	Use of emotional appeals, framing, and/or narratives targeted at adolescents’ emotions Use of persuasive arguments targeted at adolescents’ emotions
Communication outcomes	Emotions Awareness Motivation Action

Within each category, data-driven inductive coding will ensure that message content targeted at adolescent emotions is integrated into the narrative synthesis. The predetermined categories will be used to preorganize the analysis, while allowing for refinement of categories and themes as new insights emerge during analysis. In the second stage, tables will be created to connect the patterns and relationships among categories. The third stage of analysis, *data comparison*, involves synthesizing the similarities, inconsistencies, and variables in each category. In the fourth stage, the identified themes will be verified by cross-referencing the primary source data.

The analysis will be conducted by SHB and validated by all authors. Disagreements will be resolved through collaborative discussion among the authors. We will not conduct a critical appraisal of individual sources of evidence or assess the risk of bias across studies.

The patterns in the data will be interpreted and contextualized, informed by persuasion theory. Persuasion theory provides a conceptual lens for understanding how message content influences emotional and cognitive processing. It emphasizes message factors (emotional appeals, framing, and narratives) as determinants of persuasion and the interaction between affective responses and information processing depth (eg, heuristic vs systematic processing) [3].

## Results

**Figure 2.**
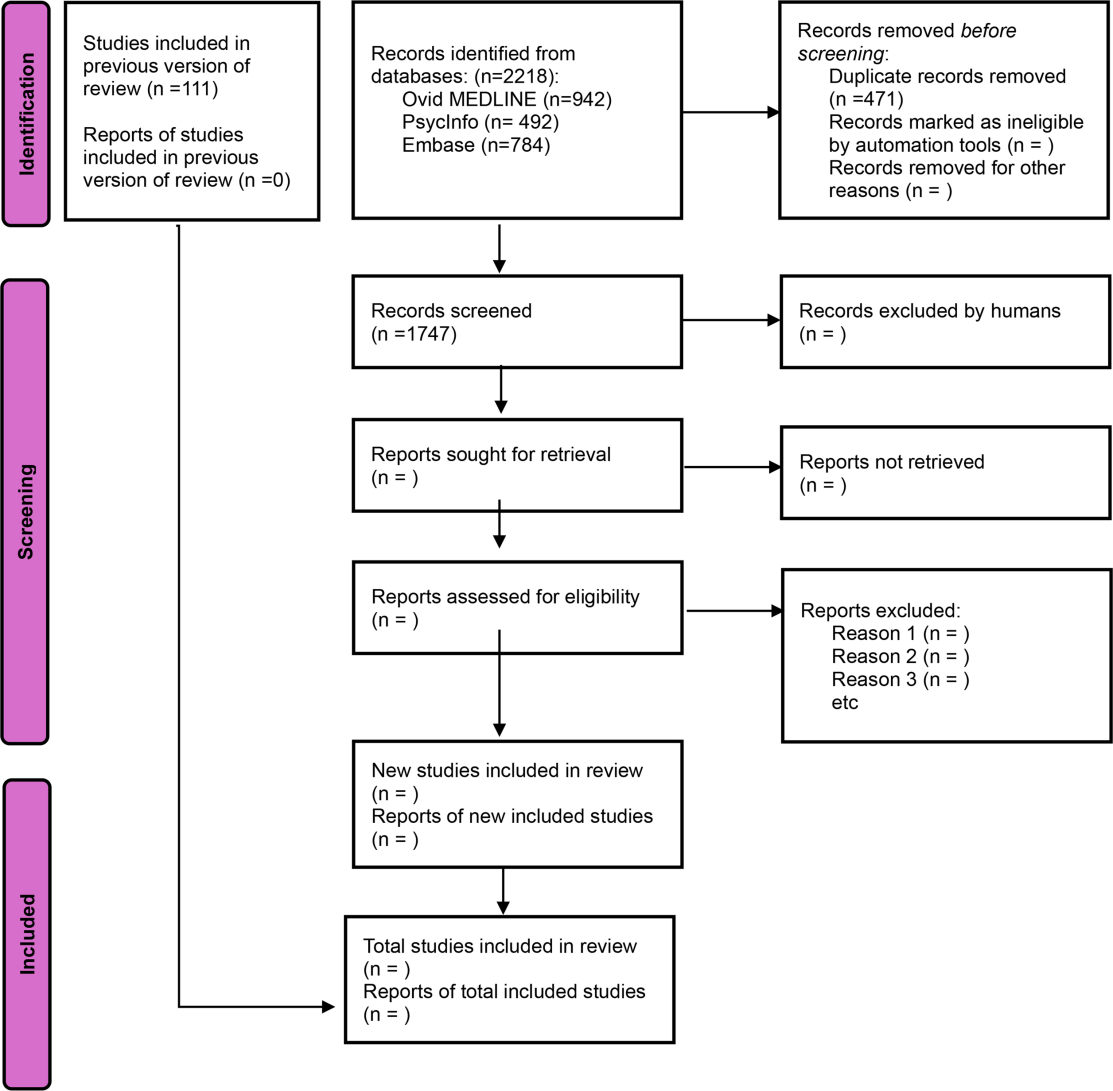
PRISMA (Preferred Reporting Items for Systematic Reviews and Meta-Analyses) flow diagram [23].

As of May 2025, the search has yielded a total of 2218 hits, of which 784 (35.3%) were from Embase, 942 (42.5%) were from MEDLINE, and 492 (22.2%) were from PsycInfo. In addition, 29 literature reviews were screened for studies sampling adolescents or young people, which resulted in 111 hits. Removing duplicates resulted in 1856 unique hits, as displayed in the PRISMA (Preferred Reporting Items for Systematic Reviews and Meta-Analyses) flow diagram (Figure 2 [23]). An updated search will be conducted in February 2026 to ensure inclusion of recent publications, and Scopus will be added to the database list to strengthen interdisciplinary coverage.

## Discussion

This review presents a synthesis of the evidence on the role of affect in health messaging toward adolescents. Through applying systematic scoping review methodology, this study will qualitatively synthesize the outcomes of the identified empirical studies using health message content targeted at adolescents’ emotions, the associated communication outcomes, and identified research gaps.

Although this review applies a descriptive synthesis, interpretation of findings will be informed by persuasion theory, which emphasizes how message factors such as emotional appeals, framing, and narratives shape both affective responses and cognitive processing depth [3]. Persuasion theory suggests that affective cues can activate heuristic or peripheral processing routes, influencing attitudes and behavioral intentions even when systematic evaluation is limited. We aim to contextualize how affective message content may lead to positive or negative outcomes among adolescents and discuss the ethical implications of using persuasive messages with this age group, whose heightened emotional sensitivity and developmental characteristics make them particularly responsive to affect-driven communication strategies.

This scoping review will provide insights into how adolescents behave, form judgments, and make decisions in response to affective health message content and highlight the communication outcomes of these studies. We anticipate finding a knowledge gap pertaining to real behavioral outcomes (actions) and that most of the literature measures intention to behave or awareness as the communication outcome.

We will discuss the strengths and limitations of the methods used in the included studies and draw attention to identified ethical challenges of communication targeted at adolescents. The scoping review findings will be discussed in comparison with previous literature on emotion and decision-making [4], health decision-making [5], health communication targeted at adolescents [10-13], and digital communication [9,14], highlighting the novel insights from this review. To conclude, we will outline policy recommendations regarding considerations that are important when designing health message content targeted at adolescents. Additionally, the review will conclude by outlining the research gaps in, for example, emotion science and communication studies and offering recommendations for future studies and campaigns in selecting theory and communication strategies.

The strength of this scoping review is the systematic application of rigorous research methodology, which strives for transparency and methodological rigor in all phases of the review process. A limitation of this scoping review is the exclusion of gray literature in the search strategy, which may bias findings toward academic and Western contexts in which peer-reviewed publications are more common. A major limitation of this review is the unclear conceptual validity of affect in message content. We address this limitation by using predefined operationalized terms, which may provide transparency regarding the study selection strategy applied and strengthen the theoretical generalization of our findings to other contexts.

## Supplementary material

10.2196/78927 Multimedia Appendix 1 Search strategy.

10.2196/78927 Checklist 1 PRISMA-ScR checklist.

## Abbreviations

MeSH: Medical Subject Headings
PRISMA: Preferred Reporting Items for Systematic Reviews and Meta-Analyses
PRISMA-ScR: Preferred Reporting Items for Systematic Reviews and Meta-Analyses extension for Scoping Reviews
