# Supplementary material for: Affective Health Messages for Adolescents: Protocol for a Scoping Review
Source: JMIR Res Protoc. 2026 Mar 2;15:e78927. doi: 10.2196/78927 (PMC12954714; doi:10.2196/78927)
Supplement: Multimedia Appendix 1 [file resprot-v15-e78927-s001.docx]

**Appendix 1. Search strategy and results**

**Ovid MEDLINE(R) ALL <1946 to April 11, 2025>**

**Number of hits: 942**

1 (Dual process theor* or dual process model or risk as feeling or heuristic cues or affect heuristic* or appraisal theor* or cognitive appraisal* or Cognitive-Experiential Self-Theory or Somatic Marker or self-efficacy or intuitive-experiental or analytical-rational thinking or system 1 thinking or system-1 thinking or information processing theor* or valence or framing* or frame or narrative* or empath* or message feature* or message appeal*).mp. [mp=title, book title, abstract, original title, name of substance word, subject heading word, floating sub-heading word, keyword heading word, organism supplementary concept word, protocol supplementary concept word, rare disease supplementary concept word, unique identifier, synonyms, population supplementary concept word, anatomy supplementary concept word] 389082

2 ((((negative or positive) adj3 (appeal* or emotion*)) or (value adj3 approach) or (presentation or communicat*)) adj3 mode*).mp. [mp=title, book title, abstract, original title, name of substance word, subject heading word, floating sub-heading word, keyword heading word, organism supplementary concept word, protocol supplementary concept word, rare disease supplementary concept word, unique identifier, synonyms, population supplementary concept word, anatomy supplementary concept word] 11340

3 emotions/ or affect/ or fear/ or anger/ or hope/ or shame/ or self-efficacy/ or heuristics/ 175976

4 1 or 2 or 3 558119

5 (digital communication or health message* or social media).mp. [mp=title, book title, abstract, original title, name of substance word, subject heading word, floating sub-heading word, keyword heading word, organism supplementary concept word, protocol supplementary concept word, rare disease supplementary concept word, unique identifier, synonyms, population supplementary concept word, anatomy supplementary concept word] 48970

6 health communication/ or social media/ or health education/ or consumer health information/ or health promotion/ 167857

7 5 or 6 195804

8 behavior/ or health behavior/ or decision making/ or judgment/ 216654

9 (decision* adj3 (make* or making*)).mp. [mp=title, book title, abstract, original title, name of substance word, subject heading word, floating sub-heading word, keyword heading word, organism supplementary concept word, protocol supplementary concept word, rare disease supplementary concept word, unique identifier, synonyms, population supplementary concept word, anatomy supplementary concept word] 373317

10 (behavior* or judgement* or behaviour*).mp. [mp=title, book title, abstract, original title, name of substance word, subject heading word, floating sub-heading word, keyword heading word, organism supplementary concept word, protocol supplementary concept word, rare disease supplementary concept word, unique identifier, synonyms, population supplementary concept word, anatomy supplementary concept word] 2215763

11 8 or 9 or 10 2544727

12 (young people or young person* or youth or adolescen* or high school*).mp. [mp=title, book title, abstract, original title, name of substance word, subject heading word, floating sub-heading word, keyword heading word, organism supplementary concept word, protocol supplementary concept word, rare disease supplementary concept word, unique identifier, synonyms, population supplementary concept word, anatomy supplementary concept word] 2479044

13 adolescent/ 2320206

14 12 or 13 2479044

15 (health* or medical*).mp. [mp=title, book title, abstract, original title, name of substance word, subject heading word, floating sub-heading word, keyword heading word, organism supplementary concept word, protocol supplementary concept word, rare disease supplementary concept word, unique identifier, synonyms, population supplementary concept word, anatomy supplementary concept word] 6637986

16 4 and 7 and 11 and 14 and 15 1645

17 limit 16 to yr="2014 -Current" 942

**APA PsycInfo <2002 to April 2025 Week 1>**

**Number of hits: 492**

1 (Dual process theor* or dual process model or risk as feeling or heuristic cues or affect heuristic* or appraisal theor* or cognitive appraisal* or Cognitive-Experiential Self-Theory or Somatic Marker or self-efficacy or intuitive-experiental or analytical-rational thinking or system 1 thinking or system-1 thinking or information processing theor* or valence or framing* or frame or narrative* or empath* or message feature* or message appeal*).mp. [mp=title, abstract, heading word, table of contents, key concepts, original title, tests & measures, mesh word] 242527

2 ((((negative or positive) adj3 (appeal* or emotion*)) or (value adj3 approach) or (presentation or communicat*)) adj3 mode*).mp. [mp=title, abstract, heading word, table of contents, key concepts, original title, tests & measures, mesh word] 6570

3 emotions/ or affect/ or fear/ or anger/ or hope/ or shame/ or self-efficacy/ or heuristics/ 110809

4 1 or 2 or 3 319100

5 health communication/ or health promotion/ or health education/ or social media.mp. [mp=title, abstract, heading word, table of contents, key concepts, original title, tests & measures, mesh word] 69838

6 (digital communication or health message* or health communication or social media).mp. [mp=title, abstract, heading word, table of contents, key concepts, original title, tests & measures, mesh word] 38650

7 5 or 6 74199

8 health behavior/ or adolescent behavior/ or judgment/ or decision making/ 129121

9 (decision* adj3 (make* or making*)).mp. [mp=title, abstract, heading word, table of contents, key concepts, original title, tests & measures, mesh word] 151819

10 (behavior* or judgement* or behaviour*).mp. [mp=title, abstract, heading word, table of contents, key concepts, original title, tests & measures, mesh word] 1113470

11 8 or 9 or 10 1219377

12 (teen* or young people or young person* or youth or adolescen* or high school*).mp. [mp=title, abstract, heading word, table of contents, key concepts, original title, tests & measures, mesh word] 501003

13 adolescent/ 0

14 12 or 13 501003

15 (health* or medical*).mp. [mp=title, abstract, heading word, table of contents, key concepts, original title, tests & measures, mesh word] 1112041

16 4 and 7 and 11 and 14 and 15 783

17 limit 16 to yr="2014 -Current" 492

**Embase <1974 to 2025 April 11>**

**Number of hits: 784**

1 (Dual process theor* or dual process model or risk as feeling or heuristic cues or affect heuristic* or appraisal theor* or cognitive appraisal* or Cognitive-Experiential Self-Theory or Somatic Marker or self-efficacy or intuitive-experiental or analytical-rational thinking or system 1 thinking or system-1 thinking or information processing theor* or valence or framing* or frame or narrative* or empath* or message feature* or message appeal*).mp. [mp=title, abstract, heading word, drug trade name, original title, device manufacturer, drug manufacturer, device trade name, keyword heading word, floating subheading word, candidate term word] 476973

2 ((((negative or positive) adj3 (appeal* or emotion*)) or (value adj3 approach) or (presentation or communicat*)) adj3 mode*).mp. [mp=title, abstract, heading word, drug trade name, original title, device manufacturer, drug manufacturer, device trade name, keyword heading word, floating subheading word, candidate term word] 14709

3 emotion/ or affect/ or fear/ or anger/ or hope/ or shame/ or heuristics/ or narrative.mp. [mp=title, abstract, heading word, drug trade name, original title, device manufacturer, drug manufacturer, device trade name, keyword heading word, floating subheading word, candidate term word] 374714

4 1 or 2 or 3 747782

5 social media/ or mass medium/ or mass communication/ or medical information/ 188694

6 (digital communication or health message* or social media or health communication).mp. [mp=title, abstract, heading word, drug trade name, original title, device manufacturer, drug manufacturer, device trade name, keyword heading word, floating subheading word, candidate term word] 84634

7 5 or 6 205657

8 health behavior/ or adolescent behavior/ or decision making/ 406336

9 (decision* adj3 (make* or making*)).mp. [mp=title, abstract, heading word, drug trade name, original title, device manufacturer, drug manufacturer, device trade name, keyword heading word, floating subheading word, candidate term word] 695601

10 (behavior* or judgement* or judgment or behaviour*).mp. [mp=title, abstract, heading word, drug trade name, original title, device manufacturer, drug manufacturer, device trade name, keyword heading word, floating subheading word, candidate term word] 2649175

11 8 or 9 or 10 3230785

12 (teen* or young people or young person* or youth or adolescen* or high school*).mp. [mp=title, abstract, heading word, drug trade name, original title, device manufacturer, drug manufacturer, device trade name, keyword heading word, floating subheading word, candidate term word] 2157339

13 adolescent/ 1923297

14 12 or 13 2157339

15 (health* or medical*).mp. [mp=title, abstract, heading word, drug trade name, original title, device manufacturer, drug manufacturer, device trade name, keyword heading word, floating subheading word, candidate term word] 9995777

16 4 and 7 and 11 and 14 and 15 1031

17 limit 16 to yr="2014 - 2024" 784
